# Hormonal Treatment Strategies Tailored to Non-Binary Transgender Individuals

**DOI:** 10.3390/jcm9061609

**Published:** 2020-05-26

**Authors:** Carlotta Cocchetti, Jiska Ristori, Alessia Romani, Mario Maggi, Alessandra Daphne Fisher

**Affiliations:** 1 Andrology, Women’s Endocrinology and Gender Incongruence Unit, Florence University Hospital, 50139 Florence, Italy; carlotta.cocchetti@gmail.com (C.C); jiska.ristori@unifi.it (J.R.); alessiaromani@hotmail.it (A.R.); 2 Department of Experimental, Clinical and Biomedical Sciences, Careggi University Hospital, 50139 Florence, Italy; m.maggi@dfc.unifi.it

**Keywords:** non-binary, transgender, gender-affirming hormonal treatment, personalized medicine

## Abstract

Introduction: To date no standardized hormonal treatment protocols for non-binary transgender individuals have been described in the literature and there is a lack of data regarding their efficacy and safety. Objectives: To suggest possible treatment strategies for non-binary transgender individuals with non-standardized requests and to emphasize the importance of a personalized clinical approach. Methods: A narrative review of pertinent literature on gender-affirming hormonal treatment in transgender persons was performed using PubMed. Results: New hormonal treatment regimens outside those reported in current guidelines should be considered for non-binary transgender individuals, in order to improve psychological well-being and quality of life. In the present review we suggested the use of hormonal and non-hormonal compounds, which—based on their mechanism of action—could be used in these cases depending on clients’ requests. Conclusion: Requests for an individualized hormonal treatment in non-binary transgender individuals represent a future challenge for professionals managing transgender health care. For each case, clinicians should balance the benefits and risks of a personalized non-standardized treatment, actively involving the person in decisions regarding hormonal treatment.

## 1. Introduction

Gender identity is an aspect of the internal sense of self of each individual, which can be masculine, feminine, a mix of both or neither [1]. For most people, the assigned sex at birth is congruent with gender identity and we refer to these individuals as cisgender. On the other hand, transgender is an umbrella term that describes the broad spectrum of individuals who transiently or persistently identify with a gender different from their natal gender [2,3].

In many cultures gender identity has been looked at through a binary lens with only two possible choices: male or female. This conceptualization takes the name of gender binarism, according to which biological sex and gender identity are categorized in two mutually exclusive forms of masculine and feminine, following the supposed dualism existing in nature. The binary conception of gender has been extended also to the transgender experience, describing an identification with the opposite gender as the only possible option for people with gender incongruence (i.e., gender dysphoria according to the DSM 5, Diagnostic and statistical manual of mental disorders) [3]. Accordingly, the term transwomen has been traditionally used to describe individuals assigned male at birth (AMAB) who identify as women, whereas transmen has been used for individuals assigned female at birth (AFAB) who identify as men.

In Western clinical models, gender identity has been categorized according to this binary concept. In line with that, in recent years biomedical research has mainly focused on the validation of standardized gender-affirming hormonal treatments capable of inducing nearly complete masculinization or feminization. Indeed, the two major goals of gender-affirming hormonal treatment were classically employed to (i) suppress endogenous sex hormone secretion, as determined by the person’s genetic/gonadal sex, and (ii) maintain sex hormone levels within the physiologic range for the person’s affirmed gender [4].

However, the traditional view of gender binarism has started to be questioned by several epidemiological studies highlighting a significant prevalence (1.9–2.8%) of gender non-conformity [5,6] in the general population (although gender non-conformity does not necessarily imply a non-binary gender identity). This aspect has been specifically assessed in a recent Dutch study [7] exploring gender identity in a group of 415 transgender individuals. The study revealed a high prevalence of non-binary gender identity (18.3%) in the evaluated sample. According to this dimensional perspective, professionals dealing with transgender health must ensure individualized and flexible interventions during a gender-affirming path, when this is requested [2,4]. This recommendation also includes hormonal treatments that should be tailored as much as possible to the person’s needs, beyond the dichotomic goals of a traditional binary model [8]. In particular, AFAB transgender individuals may request partial masculinization or AMAB transgender individuals may desire partial femininization, possibly without affecting sexual function [9]. At the same time, non-binary transgender individuals may require gender-affirming surgery (i.e., mastectomy in AFAB transgender individuals) before the start of hormonal treatment.

To date, there are no evidence-based protocols for standardized treatment in non-binary individuals and the correlated long-term risks of these regimens remain unknown. Furthermore, possible treatment strategies in non-binary transgender individuals have not yet been explored in the current literature. In line with that, the aims of the present review are (i) to suggest for the first time treatment strategies for non-binary transgender individuals with non-standardized requests and (ii) to emphasize the importance of a personalized clinical approach. In order to respond to individualized and different clients’ requests, clinicians should combine different hormonal formulations, bearing in mind their mechanism of action, efficacy and safety.

## 2. Methods

The methodology used in this narrative review consisted of a careful analysis of literature regarding hormonal and non-hormonal compounds which could be used in non-binary transgender individuals. We considered both review and original articles regarding different compounds’ categories, focusing on studies conducted on the transgender population. A computerized search was performed independently by two authors using PubMed. Search terms included gender-affirming hormonal treatment, non-binary, transgender, anti-androgens, hirsutism treatment, androgens, estrogens, androgenic-anabolic steroids and gonadotropin-releasing hormone agonists (GnRHa). In addition, reference sections of the identified articles were evaluated for further relevant publications. Only English publications were selected. Studies resulting from the aforementioned search terms were selected in accordance with the aims of the present review and the clinical interest for the reader.

## 3. Hormonal Compounds

### 3.1. Estrogens

**Figure 1:**
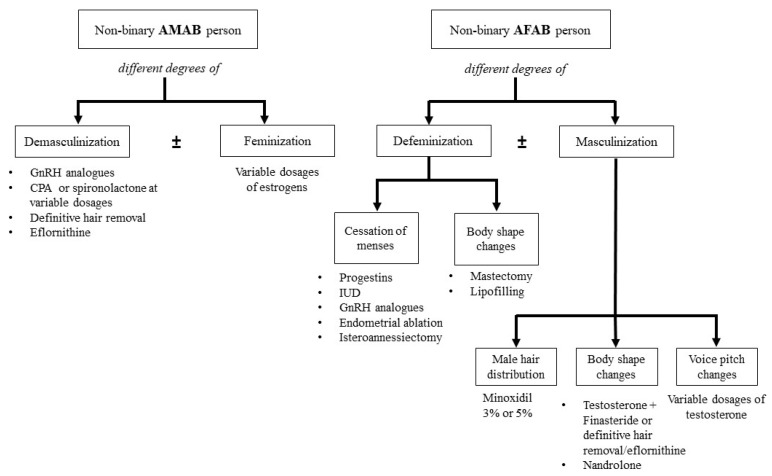
Flowchart reporting possible treatment options in non-binary transgender individuals. The figure includes hormonal/non-hormonal compounds and other strategies (i.e., procedures and surgical interventions) that could be proposed to non-binary transgender individuals on the basis of their requests. Variable dosages of hormonal compounds are discussed in the text. AMAB: assigned male at birth; AFAB: assigned female at birth; GnRH: gonadotropin-releasing hormone CPA: cyproterone acetate; IUD: intrauterine device.

In AMAB transgender individuals, estrogens are required to induce desired female secondary sexual characteristics. Among available compounds, 17-β-estradiol represents the most commonly used, since the synthetic estrogen ethinyl estradiol has been associated with increased cardiovascular and thromboembolic risks [10,11]. The common dosage of estrogen during gender-affirming hormonal treatment is two or three times as high as the recommended doses for hormone replacement therapy in postmenopausal women, with a goal of serum estradiol and testosterone of 100–200 pg/mL (or 367–734 pmol/L) and less than 50 ng/dL (or 1.7 nmol/L), respectively [4]. The aforementioned goals are those requested by trans women who want to achieve a complete feminization with suppression of male sexual characteristics. For this reason, in non-binary AMAB individuals it could be necessary to adjust these goals depending on patients’ requests. On the other hand, non-binary AMAB individuals requesting full feminization (i.e., breast development, body composition, skin tenderness) and preservation, at the same time, of erectile function can benefit from only-estrogens treatment or estrogens combined with low dosages of cyproterone acetate (10mg/daily or 25 mg on alternative days) or with 5α-reductase inhibitors (Figure 1).

### 3.2. Androgens

In AFAB transgender individuals, testosterone is used to induce virilizing effects. Among available compounds, the most commonly prescribed are injectable testosterone esters, long acting testosterone undecanoate and testosterone gel and patches, with no differences regarding short-term safety and satisfaction [12]. In binary AFAB people requesting complete virilization, regimens of testosterone treatment follow the general principle of hormone replacement treatment of male hypogonadism, with the aim to achieve testosterone values in the normal male range (generally 320 to 1000 ng/dL or 11 to 34.7 nmol/L) [4]. However, in non-binary AFAB individuals requesting partial masculinization, it may be possible to adjust the dose of testosterone or add other hormonal preparations in order to model the effects of androgens on the body (Figure 1). When the main goal is represented by body composition changes (i.e., muscle mass increase) and voice deepening, but facial and body hair increase is not desired, testosterone treatment can be combined with 5α-reductase inhibitors or definitive hair removal. In fact, in the hair follicle, testosterone is converted by 5α-reductase enzyme into 5α-dihydro-testosterone (DHT), which subsequently regulates dermal physiology through intracrine and paracrine manners [13]. Finasteride, by inhibiting 5α-reductase type 2, interferes with DHT action and results in effective treatment of androgenetic alopecia, thus its use (1 mg/daily) could be extended to this application. Furthermore, several studies conducted on cisgender women have shown that finasteride is as effective as spironolactone or flutamide in treating hirsutism, due to the ability to block the conversion of testosterone into DHT in the hair follicle [14].

Other options may include nandrolone, an anabolic steroid administered via intramuscular injection, which is not as optimal a substrate for 5α-reductase as testosterone, but it has a stronger effect compared to the testosterone on target tissues devoid of 5α-reductase activity (e.g., muscular tissue) [15]. Indeed, nandrolone can be theoretically used in non-binary AFAB individuals requesting masculinization of body shape (i.e., increased muscle mass) with a limited increase in facial and body hair. Regarding the safety profile of this compound, data are limited by the fact that most observations come from the setting of androgenic-anabolic steroid (AAS) abuse [16,17], thus their applicability to appropriate medical therapy is limited [18]. In this setting, concerns about cardiomyopathy and coronary artery disease risk emerged [19], although associated with the administration of nandrolone at extremely higher dosages [20]. Furthermore, nandrolone use does not seem associated to hepatotoxicity, since, as an injectable oil, it is not subject to first-pass hepatic metabolism.

### 3.3. Progestogens

Historically, progestogens have been used as additional antiandrogenic treatment, since they feed back to the hypothalamus lowering luteinizing hormone (LH) and testosterone levels and compete for the 5α-reductase enzyme, which converts testosterone into DHT [21]. Moreover, some trans women may request progesterone to enhance breast development. A recent opinion [22] suggests the use of oral micronized progesterone in trans women in order to endorse feminizing effects during gender-affirming hormonal treatment, possibly by promoting ductal branching within the breast [23], and to prevent negative long-term cardiovascular and bone effects. However, to date there are no clinical studies that support a positive effect of progestogens on breast development in transgender persons [24]. Current guidelines [4] recommend against routine progestogen use in gender transition. In fact, evidence from studies conducted on cisgender women taking progesterone suggests an increased risk of thromboembolism and stroke [25,26]. Finally, progestogens may induce water retention leading to weight gain and elevation of blood pressure [27].

Apart from this, progestins, such as lynestrenol 5–10 mg/daily, medroxyprogesterone acetate 5–10 mg/daily or norethindrone 15 mg/daily, can find application in AFAB transgender individuals to stop menses when testosterone alone is not sufficient [4,28]. Additionally, progestins can be used for non-binary AFAB individuals reporting high distress towards menses but requesting none or partial masculinization. For this purpose, progestin agents can be used alone or in combination with low doses of testosterone. Alternative options to reach this goal are represented by GnRH agonists, endometrial ablation [4,29] or, especially when reversible contraception is desired, a progesterone-releasing intrauterine device (IUD) [30] (Figure 1).

### 3.4. Other Androgen Lowering Therapies

Antiandrogens include different compounds able to inhibit androgen secretion or action, mainly used in AMAB transgender people in order to reduce typical male characteristics and to attain required serum estradiol levels.

Cyproterone acetate (CPA) is one of most common antiandrogens used in Europe. CPA competes with androgens for binding to the androgen receptor (AR) and inhibits adrenal steroidogenesis [31]. In addition, it exerts a negative hypothalamic feedback through its progestogenic properties, leading to a reduction of testicular androgen secretion. Traditionally, the recommended dose of CPA for AMAB transgender people ranged from 50 to 100 mg/daily [4]. Since CPA is highly lipophilic, it accumulates in the subcutaneous adipose tissue, and therefore, a lower dose or administration on alternate days can be used to reduce androgen levels [32]. The main critical issues regarding the use of CPA concern its hepatotoxicity and the potential increased risk of meningiomas, which are hormone-sensitive tumours expressing progesterone receptors [33,34]. However, in the transgender population, only a transient elevation of liver enzymes has been reported [35], and no significant differences have been observed between trans women under hormonal treatment and those not receiving gender-affirming hormones [36].

Spironolactone (100–300 mg/daily) is commonly prescribed in the United States, where CPA is not available. Spironolactone is an antagonist of the mineralocorticoid receptor with antiandrogenic properties, mainly exerted by blocking the AR [37]. A recent study [38] demonstrated that spironolactone 200 mg daily is effective in reducing testosterone levels in AMAB transgender individuals into the cisgender female range, with an interval of nine months required to reach a steady-state of testosterone. The heterogeneity of this effect was mainly explained by the body mass index (BMI), with individuals with a lower BMI having higher testosterone levels. However, this evidence may be limited by the co-administration of estradiol in the whole sample. Besides, other authors did not find any effect of spironolactone on testosterone levels [39]. Furthermore, spironolactone use is limited by the risk of hyperkalemia, hypotension and gastrointestinal bleeding [40].

Other options may include nonsteroidal antiandrogens, such as flutamide (50–75 mg/daily) and bicalutamide (25–50 mg/daily), which block the AR and, therefore, reduce androgens action. On the other hand, these compounds increase gonadotropin secretion, compromising the reduction of circulating testosterone levels observed with steroidal antiandrogens. The lack of data about their efficacy and safety in the transgender population, as well as the high risk of hepatotoxicity described in ciswomen, do not allow their use to be recommended [41].

Among the 5α-reductase inhibitors, finasteride and dutasteride can be used in selected cases leveraging their capacity to inhibit the conversion of testosterone into 5α-dihydro-testosterone (DHT). Even if some concerns related to sexual dysfunction and depression risk are reported in the literature in cisgender men [42,43], their efficacy and safety on treatment in this population of androgenetic alopecia has been reported [44,45], thus they could be considered to stop male pattern hair loss in binary AMAB transgender individuals.

GnRHa, such as triptorelin, leuprorelin and goserelin, represent an effective alternative to reduce testosterone levels, through the down-regulation of GnRH receptor in the pituitary. They are considered extremely safe, although their use is limited by the high cost. Potential new alternatives to GnRHa are GnRH antagonists, which do not have the criticality of the initial “flare” in gonadotropic axis activation, but their use in transgender population is still limited [4].

Androgen lowering compounds can be included among hormonal treatment strategies in both non-binary AFAB and AMAB people (Figure 1). Indeed, in the case of AMAB agender persons—wishing only to attenuate masculine characteristics, without inducing any feminization—only nonsteroidal or steroidal anti-androgens can be considered. The choice between decreasing androgen production (with steroidal antiandrogens) or only androgen peripheral action (with nonsteroidal ones) may be based on individual phenotypical goals. As androgen-deprivation therapy results in a deleterious effect on bone mineral density [46], a lower dosage of estrogens can be discussed with clients. However, no data are available regarding long-term critical effects when estrogen levels during gender-affirming hormonal treatment do not reach the usual therapeutic goal for binary transgender individuals. Theoretically, nonsteroidal antiandrogens do not compromise estrogen synthesis, because testosterone levels are high and aromatase activity is still efficient. Moreover, some AFAB transgender individuals can benefit from testosterone therapy combined with 5α-reductase inhibitors or from treatment with nandrolone (an androgenic compound less prone to 5α reduction) in case they wish only a partial virilization (i.e., voice deepening and lean mass increase without facial and body hair increase).

## 4. Non-Hormonal Compounds and Other Strategies

Several non-hormonal compounds can be prescribed to modulate the effects of hormonal treatment in non-binary transgender individuals.

Minoxidil is a topical treatment approved for androgenetic alopecia, available at 2% and 5% solution preparations. This drug is a peripheral vasodilator, opening potassium channels located on the smooth muscles of the peripheral artery [47]. Through its metabolite, minoxidil sulfate, it stimulates the growth of follicle keratinocytes and prolongs the anagen phase [48]. Although it is considered to be safe and beneficial, the most common reported side effects of minoxidil are irritant contact dermatitis and hypertrichosis. Topical application did not generally show systemic effects such as hypotension and abnormal heart rate [49]. Minoxidil is generally used topically twice per day in the treatment of androgenetic alopecia. For this kind of application, it can be prescribed in AFAB transgender people experiencing androgenetic alopecia during treatment with conventional doses of testosterone [50]. Moreover, minoxidil lotion (3%) has also been demonstrated superior to placebo in significantly increasing hair count in cisgender population after sixteen weeks of treatment [51]. Thus, its application can be recommended as an additional treatment to testosterone in binary AFAB transgender people to promote beard growth. In addition, minoxidil application can be suggested to non-binary AFAB individuals desiring beard development but not (or not complete) body male shaping (e.g., lean mass increase, voice pitch changes).

Topical eflornithine reduces the rate of hair growth through the irreversible inhibition of ornithine decarboxylase, an enzyme which is essential for follicular polyamine synthesis. Eflornithine usually takes from six to eight weeks to give noticeable results and is a lifelong therapy, since hair growth resumes once treatment is discontinued [52]. For this reason, eflornithine is generally used in conjunction with other therapies. Indeed, two randomized controlled trials demonstrated that eflornithine can improve the efficiency of standard laser hair removal in cisgender people [53,54]. Eflornithine’s topical use, combined with oral anti-androgens and laser hair removal, can further promote hair removal in binary AMAB transgender people. Moreover, eflornithine can be offered as a treatment to reduce facial and body hair in non-binary transgender individuals not wanting sexual function impairment or body feminization, which usually result from traditional hormonal treatment (with antiandrogens or estrogens).

Furthermore, permanent methods of hair reduction may be proposed to AMAB transgender people. These include photoepilation, which is capable of treating large areas but requires the presence of pigmented terminal hair, and electrolysis, generally used to treat small areas regardless of hair pigmentation [52]. Similarly to eflornithine, these options can be discussed with non-binary AMAB individuals desiring only hair reduction.

Finally, in case the available compounds do not allow the desired goals to be achieved, surgical interventions can be proposed. Mastectomy and lipofilling may represent an option for non-binary AFAB individuals requesting body shape changes without masculinization, while breast augmentation can be offered to non-binary AMAB individuals, eventually in addition to low estrogen dosages.

## 5. Conclusions

Non-binary transgender individuals represent a growing body of clients referring to specialized gender clinics. In fact, requests for non-standardized hormonal treatments are increasing. In the near future, clinicians dealing with transgender health care will have to face these requests more frequently and be aware of the need to implement truly individualized hormonal treatment. On the other hand, there is a lack of data in the literature regarding possible therapeutic strategies and their efficacy and safety. For this reason, our goal was to provide a guide for clinicians in order to better meet the requests of non-binary transgender individuals. Clinicians must bear in mind that these treatment strategies are currently not included in the international guidelines [2,4], but they may play a crucial role in better responding to non-binary transgender individuals’ needs and therefore reduce distress and improve quality of life. It will be important to define the necessary biochemical thresholds of sex hormones to achieve the desired sexual characteristics and to avoid adverse effects related to hypogonadism. In fact, to date, this information is scarce in the available literature [55]. For all these reasons, it is important that future research will focus on the specific health issues in this population.
